# Comparative Cell Surface Proteomic Analysis of the Primary Human T Cell and Monocyte Responses to Type I Interferon

**DOI:** 10.3389/fimmu.2021.600056

**Published:** 2021-02-08

**Authors:** Lior Soday, Martin Potts, Leah M. Hunter, Benjamin J. Ravenhill, Jack W. Houghton, James C. Williamson, Robin Antrobus, Mark R. Wills, Nicholas J. Matheson, Michael P. Weekes

**Affiliations:** ^1^ Cambridge Institute for Medical Research, University of Cambridge, Cambridge, United Kingdom; ^2^ Department of Medicine, University of Cambridge, Addenbrooke’s Hospital, Cambridge, United Kingdom; ^3^ Cambridge Institute of Therapeutic Immunology & Infectious Disease (CITIID), University of Cambridge, Cambridge, United Kingdom; ^4^ NHS Blood and Transplant, Cambridge, United Kingdom

**Keywords:** type I interferon (IFN), leukocytes, quantitative proteomics, cell surface, antiviral restrictin, monocyte, T cell, plasma membrane

## Abstract

The cellular response to interferon (IFN) is essential for antiviral immunity, IFN-based therapy and IFN-related disease. The plasma membrane (PM) provides a critical interface between the cell and its environment, and is the initial portal of entry for viruses. Nonetheless, the effect of IFN on PM proteins is surprisingly poorly understood, and has not been systematically investigated in primary immune cells. Here, we use multiplexed proteomics to quantify IFNα2a-stimulated PM protein changes in primary human CD14+ monocytes and CD4+ T cells from five donors, quantifying 606 and 482 PM proteins respectively. Comparison of cell surface proteomes revealed a remarkable invariance between donors in the overall composition of the cell surface from each cell type, but a marked donor-to-donor variability in the effects of IFNα2a. Furthermore, whereas only 2.7% of quantified proteins were consistently upregulated by IFNα2a at the surface of CD4+ T cells, 6.8% of proteins were consistently upregulated in primary monocytes, suggesting that the magnitude of the IFNα2a response varies according to cell type. Among these differentially regulated proteins, we found the viral target Endothelin-converting enzyme 1 (ECE1) to be an IFNα2a-stimulated protein exclusively upregulated at the surface of CD4+ T cells. We therefore provide a comprehensive map of the cell surface of IFNα2a-stimulated primary human immune cells, including previously uncharacterized interferon stimulated genes (ISGs) and candidate antiviral factors.

## Introduction

Interferons (IFN) were discovered in 1957 and have since become recognized as key components of antiviral immunity in almost all cell types (1). Early detection of viruses by cellular receptors triggers synthesis of type I (α/β) IFNs, which signal *via* the ubiquitously expressed type I IFN receptor (IFNAR). There are 12 subtypes of IFNα expressed in humans, which have a similar structure, highly conserved protein sequence and bind the same receptor. Differences are thought to arise at the level of receptor binding affinity, which may lead to variation in the magnitude of stimulation of target IFN-stimulated genes (ISGs) (2, 3). The canonical signaling pathway includes activation of JAK-STAT proteins, culminating in the transcription of an array of ISGs, many of which exhibit antiviral function. IFNs also orchestrate adaptive immune responses, enhancing T, B and natural killer cell function in addition to both positively and negatively regulating the IFN response itself (4).

Susceptibility to viral infection and disease is determined in part by certain ISGs, the IFN-stimulated antiviral restriction factors (ARFs) (5). However, different subsets of ISGs can be induced in different cell types (6, 7), partly determined by the density of IFN receptor expression, together with the pattern of expression of kinases, STAT proteins and transcription factors (8–10). Detailed characterization of the IFN response at the level of individual cell types is thus essential, and the study of primary as opposed to cultured cells has been instrumental in revealing novel facets of antiviral immunity (11–13). Primary CD4+ T cells and monocytes are particularly important, since they can not only be infected by viruses, but also play key roles in immunity to pathogens. Small-molecule disruption of the interaction between ARFs and viral antagonists can enable endogenous inhibition of viral replication (14), so identification and characterization of novel ARFs in these cells may facilitate the development of new antiviral therapies.

As well as the antiviral response, IFNs are critical for the pathogenesis of autoimmune diseases including systemic lupus erythematosus and interferonopathies such as Aicardi-Goutieres syndrome (15). In addition, IFNα2 has been used to treat cancers, multiple sclerosis and certain viral infections, including chronic hepatitis B and coronavirus disease 2019 (COVID-19) (16–20). Treatment may be accompanied by significant side-effects, and identifying candidate biomarkers to predict the likelihood of response or adverse effects from IFNα therapy would rationalize individualized therapy (21). IFNα2a was therefore selected for this study both as a representative of the IFNαs, and due to its relevance to currently employed therapeutics.

While previous studies have examined the effects of IFN stimulation in primary leukocytes at the transcriptomic level (6, 22), the correlation between transcript and protein abundance is often poor (23–25). Proteomic investigations in these cells have been limited by the available technology; for example, two-dimensional gel electrophoresis detected only seven proteins differentially expressed upon IFN stimulation of activated CD4+ T cells (26). Beyond the whole cell proteome, the PM represents a critical interface between the cell and its environment, and is the site of many drug targets. However, there have been no prior investigations of IFN-mediated protein changes at the PM.

We previously developed ‘plasma membrane profiling’ to enable quantitative investigation of cell surface proteomic changes in response to viral infection (27, 28). In this study, we adapt this technology to characterize the effects of IFNα2a at the surface of primary monocytes and CD4+ T cells. By multiplexing analysis using tandem mass tag (TMT)-based triple-stage mass spectrometry, we provide a comprehensive assessment of IFN-stimulated and unstimulated samples from five donors, including quantification of 606 and 482 annotated PM proteins in primary CD14+ monocytes and CD4+ T cells respectively. We further show how the effect of IFNα2a varies markedly between cell types and individuals, and identify ECE1 as a novel cell type-specific IFN-stimulated factor.

## Materials and Methods

### Primary Cell Isolation and Cell Culture

#### Primary Cell Isolation and Extraction for Proteomics

PBMCs were isolated by Ficoll density gradient centrifugation, from leukocyte cones purchased from the national health service blood and transplant service (NHSBT) in the case of monocytes, and peripheral blood for enrichment of CD4+ T cells. Enrichment of particular cell types from the PBMCs was by negative selection using the MACS Monocyte Isolation Kit II (Miltenyi Biotec, 130-091-153), Pan Monocyte Isolation Kit (Miltenyi Biotec, 130-096-537) and Dynabeads Untouched Human CD4 T Cells kit (Invitrogen, 11346D), according to the manufacturer’s instructions.

Monocytes and pan-monocytes were cultured overnight in X-Vivo 15 serum-free hematopoietic cell medium (Lonza), and CD4+ T cells were cultured in RPMI-1640 medium (Sigma) supplemented with 10% human AB serum (Sigma). The cells were either left unstimulated or treated with 1600 IU/ml IFNα2a for the CD14+ monocytes, or 1,000 IU/ml IFNα2a for pan-monocytes and CD4+ T cells. A very similar breadth and depth of response to IFNα2a stimulation was seen in whole pan-monocyte samples (1,000 IU/ml) compared to CD14+ monocytes (1,600 IU/ml) (**Figure S4A**), and use of criteria based on identifying proteins modulated >1 SD above or below mean fold change as opposed to enforcing a single absolute fold change across all cell types should mitigate effects due to different IFN concentrations. All cells were cultured at 37˚C in 5% CO2.

IFNα2a for the proteomic experiments and some validation was purchased from Reagent Proteins, a division of Pfenex Inc (catalog number BCA-309). Additional IFNα2a for further validation experiments was purchased from PBL Assay Science (catalog number 11100-1). In both cases, the recombinant protein was produced in E. coli, and the gene obtained from human leukocytes, with the specific activity determined by the supplier. In the case of the Reagent Protein IFN, activity was determined in a viral resistance assay using bovine kidney MDBK cells. Purity was >98% as determined by RP-HPLC and SDS-PAGE. For IFN from PBL, activity was measured using a cytopathic inhibition assay on MDBK cells with Vesicular Stomatis Virus, and on human lung adenocarcinoma cell line A549 with encephalomyocarditis virus. Purity was >95% by SDS-PAGE stained by Coomassie Blue. No further additional tests for LPS or other contaminants were detailed by the suppliers.

#### Primary Cell Isolation and Extraction for Validation Experiments

For the validation experiments shown in **Figures 5B, C, F, G, J**, primary cell enrichment and culture was as described above. For additional experiments in **Figures 5E, H**, and **Figures S5B** and **S7**, primary cells were enriched as follows. PBMCs were isolated from venous blood samples by density gradient centrifugation using Histopaque-1077 Hybri-Max (Sigma). Cells were isolated from donor PBMCs using MACS CD14 MicroBeads (Miltenyi, 130-050-201) or MACS CD4 T cell Isolation Kit (Miltenyi, 130-096-533), and an AutoMACS Pro Separator. For **Figure S5A**, CD4 isolation was carried out using CD4 MicroBeads (Miltenyi, 130-045-101). Both CD14+ monocytes and CD4+ T cells were incubated in X-Vivo 15 media.

#### Ethical Approval

For experiments relating to **Figures 5E, H** and **Figures S5**, **S7**, donors were recruited locally following informed consent with the ethical approval from the Cambridge Central Research Ethics Committee REC reference (97/092). For all other experiments ethical approval was granted by the University of Cambridge Human Biology Research Ethics Committee (HBREC.2016.011) and written informed consent was obtained from volunteers prior to blood donations.

#### Cultured Cell Lines

THP-1, Jurkat and SUPT1 cells were maintained in RPMI-1640 medium (Sigma) supplemented with 10% FBS. All cells were cultured at 37°C in 5% CO2. THP-1 cells were kindly provided by Professor Paul Lehner (Department of Medicine, University of Cambridge). All IFN stimulation of cultured cell lines was with 1,000 IU/ml IFNα2a.

### Plasma Membrane Enrichment and Proteomics

#### Plasma Membrane Enrichment

Plasma membrane profiling was performed as described previously (29, 30). Briefly, cells were centrifuged to collect and washed twice with ice-cold PBS with MgCl_2_ and CaCl_2_ (Sigma). An oxidation/biotinylation mix comprising 1 mM sodium meta-periodate (Thermo), 100 mM aminooxy-biotin (Biotium) and 10 mM aniline (Acros Organics) in ice-cold PBS pH 6.7 was applied. The samples were rocked for 30 min at 4°C in the dark, and then the reaction was quenched with 1 mM glycerol for 5 min on ice. Biotinylated cells were washed twice in PBS pH 7.4, centrifuged to collect, and lysed in 1% Triton X-100 lysis buffer (1% Triton, 10 mM Tris-HCL, 150 mM NaCl, cOmplete protease inhibitor, 5 mM IAA) for 30 min on ice. Nuclei were removed by centrifugation at 4°C, 13,000 g, 5 min, and this was repeated three times. Biotinylated glycoproteins were enriched with high affinity streptavidin agarose beads (Thermo) and washed extensively using a vacuum manifold and Poly-Prep columns (BioRad). Washing was initially with lysis buffer, then 0.5% SDS and then urea. Captured protein was reduced with DTT, alkylated with iodoacetamide (IAA, Sigma) and digested on-bead with trypsin (Promega) in 100 mM HEPES pH 8.5 for 3h. Tryptic peptides were collected. A prior study determined that biotinylation under identical conditions was confined to the cell surface, with no discernable labeling of either endosomes or lysosomes (31).

#### Peptide Labeling With Tandem Mass Tags

TMT reagents (0.8mg) were dissolved in 43 µl of anhydrous acetonitrile, and 5 µl was added to the peptide samples at a final concentration of 30% acetonitrile (v/v). Following incubation for 1 h at room temperature, the reaction was quenched with hydroxylamine to a final concentration of 0.05% (v/v). TMT labeled samples were combined 1:1 (THP-1 cells) and 1:1:1:1 (pan-monocytes), vacuum centrifuged to near dryness and then desalted using a StageTip (32) before analyzing a small fraction of the sample in a ‘single shot’ by LC-MS3.

For primary monocytes and CD4+ T cells, a small amount of each sample was initially labeled and combined 1:1:1:1:1:1:1:1:1:1 for a single shot, as described above. The remainder of the sample was then labeled and the amounts combined were adjusted to ensure equal loading of peptide from each sample, to avoid a requirement for excessive digital normalization. Samples were not quenched until adequate labeling had been confirmed by analysis of a single shot on the mass spectrometer, in some cases necessitating addition of more TMT reagent. For CD4+ T cells, single shot analysis was initially of stimulated and unstimulated samples derived from three donors, which was later extended to a 10-plex analysis on addition of samples from two further donors. Following single shot analysis of primary CD14+ monocytes and CD4+ T cells, TMT-labeled and combined samples were subject to C18 solid phase extraction (Sep-Pak, Waters) and SCX fractionation (see below) resulting in six fractions, in order to increase the overall number of peptides quantified. Each fraction was then desalted using a StageTip prior to analysis by LC-MS3.

Details of individual sample labeling, and mass spectrometry analyses are described in **Table S5**.

#### Offline Tip-Based Strong Cation Exchange SCX Fractionation

A protocol for solid-phase extraction based SCX peptide fractionation was previously modified for small peptide amounts (28). Briefly, 10 mg of PolySulfethyl A bulk material (Nest Group Inc) was loaded on to a fritted 200 µl tip in 100% Acetonitrile using a vacuum manifold. The SCX material was conditioned slowly with 2x 400 µl SCX buffer A (7mM KH_2_PO_4_, pH 2.65, 30% Acetonitrile), 400 µl SCX buffer B (7mM KH_2_PO_4_, pH 2.65, 350mM KCl, 30% Acetonitrile) and then 4x 400 µl SCX buffer A. Dried peptides were resuspended in 400 µl SCX buffer A and added to the tip under vacuum, with a flow rate of ~150 µl/min. The tip was then washed with 150 µl SCX buffer A. Fractions were eluted in 150 µl washes using SCX buffer at increasing K+ concentrations (10, 25, 40, 60, 90, 150mM KCl), vacuum-centrifuged to near dryness then desalted using StageTips.

#### LC-MS3

Mass spectrometry data was acquired using an Orbitrap Fusion for all experiments apart from the two initial CD14+ monocyte single shots and the 10-plex CD4 single shot, where an Orbitrap Fusion Lumos was used instead (Thermo Fisher Scientific, San Jose, CA), as detailed in **Table S5B**. In both cases, an Ultimate 3000 RSLC nano UHPLC equipped with a 300 µm ID x 5 mm Acclaim PepMap µ-Precolumn (Thermo Fisher Scientific) and a 75 µm ID x 50 cm 2.1 µm particle Acclaim PepMap RSLC analytical column was used.

For Orbitrap Fusion Lumos experiments: Loading solvent was 0.1% FA, analytical solvent A: 0.1% FA and B: 80% MeCN + 0.1% FA. Separations were carried out at 40°C (gradient 1) or 55°C (gradient 2 and 3). Samples were loaded at 5 µl/min for 5 min in loading solvent before beginning the analytical gradient. The following gradients were used. Gradient 1: 3%–40% B over 55 min, followed by a 5 min wash at 95% B and equilibration at 3% B for 10 min. Gradient 2: 3%–7% B over 3 min, 7%–37% over 54 min followed by 2 min wash at 95% B and equilibration at 3% B for 15 min. Gradient 3: 3%–7% B over 3 min, 7%–37% B over 176 min followed by a 4 min wash at 95% B and equilibration at 3% B for 15 min. Each analysis used a MultiNotch MS3-based TMT method (33). The following settings were used: MS1: 380–1,500 Th, 120,000 Resolution, 2x10^5^ automatic gain control (AGC) target, 50 ms maximum injection time. MS2: Quadrupole isolation at an isolation width of m/z 0.7, CID fragmentation (normalized collision energy (NCE) 35) with ion trap scanning in turbo mode from m/z 120, 1x10^4^ AGC target and 50 ms maximum injection time for gradient 1 or 1.5x10^4^ AGC target and 120 ms maximum injection time for gradients 2 and 3. MS3: In Synchronous Precursor Selection mode the top 10 MS2 ions were selected for HCD fragmentation (NCE 65) and scanned in the Orbitrap at 60,000 resolution with an AGC target of 1x10^5^ and a maximum accumulation time of 120 ms for gradient 1 or 150 ms for gradients 2 and 3. Ions were not accumulated for all parallelisable time. The entire MS/MS/MS cycle had a target time of 3 s. Dynamic exclusion was set to +/− 10 ppm for 70 s. MS2 fragmentation was trigged on precursors 5x10^3^ counts and above.

For Orbitrap Fusion experiments: Loading solvent was 0.1% TFA, analytical solvent A: 0.1% FA and B: MeCN + 0.1% FA. All separations were carried out at 55°C. Samples were loaded at 10 µl/min for 5 min in loading solvent before beginning the analytical gradient. All samples were run with a gradient of 3%–34% B, followed by a 5 min wash at 80% B, a 5 min wash at 90% B and equilibration at 3% B for 5 min, resulting in a total gradient over the time indicated in **Table S5B**. Each analysis used a MultiNotch MS3-based TMT method (33). The following settings were used: MS1: 400-1400 Th, Quadrupole isolation, 120,000 Resolution, 2x10^5^ AGC target, 50 ms maximum injection time, ions injected for all parallizable time. MS2: Quadrupole isolation at an isolation width of m/z 0.7, CID fragmentation (NCE 30) with ion trap scanning out in rapid mode from m/z 120, 1x10^4^ AGC target, 70 ms maximum injection time, ions accumulated for all parallizable time in centroid mode. MS3: in Synchronous Precursor Selection mode the top 10 MS2 ions were selected for HCD fragmentation (NCE 65) and scanned in the Orbitrap at 50,000 resolution with an AGC target of 5x10^4^ and a maximum accumulation time of 150 ms, ions were not accumulated for all parallelisable time. The entire MS/MS/MS cycle had a target time of 3 s. Dynamic exclusion was set to +/- 10 ppm for 90 s. MS2 fragmentation was trigged on precursors 5x10^3^ counts and above.

### Quantification and Statistical Analysis

#### Data Analysis

Mass spectra were processed using a Sequest-based software pipeline for quantitative proteomics, “MassPike”, through a collaborative arrangement with Professor Steve Gygi’s laboratory at Harvard Medical School. MS spectra were converted to mzXML using an extractor built upon Thermo Fisher’s RAW File Reader library (version 4.0.26). In this extractor, the standard mzxml format has been augmented with additional custom fields that are specific to ion trap and Orbitrap mass spectrometry and essential for TMT quantitation. These additional fields include ion injection times for each scan, Fourier Transform-derived baseline and noise values calculated for every Orbitrap scan, isolation widths for each scan type, scan event numbers, and elapsed scan times. This software is a component of the MassPike software platform and is licensed by Harvard Medical School.

The human UniProt database (26th January, 2017), was combined with a database of common contaminants such as porcine trypsin. The combined database was concatenated with a reverse database composed of all protein sequences in reversed order. Searches were performed using a 20 ppm precursor ion tolerance (34). Product ion tolerance was set to 0.03 Th. TMT tags on lysine residues and peptide N termini (229.162932 Da) and carbamidomethylation of cysteine residues (57.02146 Da) were set as static modifications, while oxidation of methionine residues (15.99492 Da) was set as a variable modification.

To control the fraction of erroneous protein identifications, a target-decoy strategy was employed (35, 36). Peptide spectral matches (PSMs) were filtered to an initial peptide-level false discovery rate (FDR) of 1% with subsequent filtering to attain a final protein-level FDR of 1% (37, 38). PSM filtering was performed using a linear discriminant analysis, as described (39). This distinguishes correct from incorrect peptide IDs in a manner analogous to the widely used Percolator algorithm (40), though employing a distinct machine learning algorithm. The following parameters were considered: XCorr, ΔCn, missed cleavages, peptide length, charge state, and precursor mass accuracy. Protein assembly was guided by principles of parsimony to produce the smallest set of proteins necessary to account for all observed peptides (39).

Proteins were quantified by summing TMT reporter ion counts across all matching peptide-spectral matches using “MassPike”, as described (33, 41). A minimum of one unique or shared peptide per protein was used for quantitation. Briefly, a 0.003 Th window around the theoretical m/z of each reporter ion (126, 127n, 127c, 128n, 128c, 129n, 129c, 130n, 130c, 131n) was scanned for ions, and the maximum intensity nearest to the theoretical m/z was used. The primary determinant of quantitation quality is the number of TMT reporter ions detected in each MS3 spectrum, which is directly proportional to the signal-to-noise (S:N) ratio observed for each ion (42). Conservatively, every individual peptide used for quantitation was required to contribute sufficient TMT reporter ions so that each on its own could be expected to provide a representative picture of relative protein abundance (41). Additionally, an isolation specificity filter with a cut-off of 50% was employed to minimize peptide co-isolation (43). Peptide-spectral matches with poor quality MS3 spectra (more than 9 TMT channels missing and/or a combined S:N ratio of less than 25 for each channel used (as detailed in **Table S5B**) or no MS3 spectra at all were excluded from quantitation. Peptides meeting the stated criteria for reliable quantitation were then summed by parent protein, in effect weighting the contributions of individual peptides to the total protein signal based on their individual TMT reporter ion yields. Protein quantitation values were exported for further analysis in Excel.

For protein quantitation, reverse and contaminant proteins were removed, then each reporter ion channel was summed across all quantified proteins and normalized assuming equal protein loading across all channels. For all TMT experiments, normalized S:N values are presented in **Table S1** (‘Data’ worksheet). Proteins were defined as being PM proteins, and used in further analysis if they had a GO annotation of “plasma membrane”, “cell surface”, “extracellular” or “short GO” (29). All analysis was performed following filtering of the data to include only proteins that had relevant gene ontology annotations.

As there are challenges in confidently assigning peptides to a specific HLA allele, and to account for different alleles being expressed in the different donors, the S:N values were summed to give a single value for HLA-A, HLA-B, HLA-C and HLA-DRB1. Additionally, all classical HLA molecules were excluded from the investigation of cell surface protein abundance (**Figure 2**, **Figure S2A**, and **Table S3**). Furthermore, data for proteins quantified in <3 donors following subsequent filtering (NOX4, GYPA, JAK3 and SLC25A3) was removed prior to analysis.

To estimate the relative abundance of each protein, a method based on iBAQ was employed. The summed MS1 maximum precursor intensity for each protein across all matching peptides was calculated. Each value was divided by the number of theoretically observable tryptic peptides 7–30 amino acids in length for the respective protein, as determined by in silico trypsin digestion of human Swissprot canonical and isoform database (2017_01_26) using the OrgMassSpecR51 package in R 3.5.152. To determine the abundance of a protein at the surface of unstimulated cells, the summed intensity was adjusted in proportion to normalized S:N values: (Donor 1 + Donor 2 + Donor 3 + Donor 4 + Donor 5 unstimulated)/∑(all donors ± IFN) (**Figure 2**). To compare donors, the intensity was adjusted in proportion to the unstimulated sample for that donor (eg Donor 1 unstimulated/∑(all donors ± IFN) (**Figure S2A**).

To prevent overestimation of the IFN-induced fold change (FC), individual donor FCs derived from S:N values that contributed < 2% of the total S:N in either the IFNα2a stimulated or unstimulated sample were excluded. Proteins were only considered to be consistently upregulated if the fold change was >1 for every donor in which the protein was quantified, and <1 in every donor for downregulation.

#### Statistical Analysis

**Figure 2C**. The percent of the cell surface contributed by each protein in monocytes and T cells was compared. The ratios were log_2_ transformed, and the mean and SD across all 280 proteins calculated, in order to determine which proteins changed > 1, 2, or 3 SD from the mean protein fold change.

**Figure 3** and **Figure S3**. P-values were calculated in Excel using a paired, two-tailed Student’s t-test on log-transformed data, then subjected to Benjamini-Hochberg multiple testing correction (44). The color of the average fold change bar was determined by log_2_ transforming the data and determining the number of standard deviations the FC for a given protein was away from the mean FC for all proteins, as indicated in the legend.

**Figure S1**. P values were estimated using significance B (45), calculated using Perseus version 1.5.4.1 (46).

**Figure S2**. r^2^ values were calculated using Excel.

### Data Availability

The mass spectrometry proteomics data have been deposited to the ProteomeXchange Consortium (http://www.proteomexchange.org/) *via* the PRIDE (47) partner repository with the dataset identifier PXD022834. All materials described in this manuscript, and any further details of protocols employed can be obtained on request from the corresponding author by email to mpw1001@cam.ac.uk.

### Cell Surface Flow Cytometry

Cells were washed in FACS buffer (PBS with 2% FBS), prior to blocking in Human Trustain (Biolegend, 422302, 1:20 in FACS buffer) for 10 min at room temperature. Samples shown in **Figure S7** were additionally blocked with mouse serum (1:50). All stains were performed for 30 min at 4°C, with antibodies diluted in FACS buffer: anti-CD14-PE (Biolegend, 301850, 1:20, RRID: AB_2564138), anti-CD4-APC (Biolegend, 317415, 1:20, RRID: AB_571944), anti-CD3-FITC (Biolegend, 300406, 1:20, RRID: AB_314060), anti-IFNAR2-APC (Miltenyi, 130-099-560, 1:20, RRID: AB_2652223), anti-BST2-PE (Biolegend, 348405, 1:20, RRID: AB_10567247), anti-CD69-PE (Biolegend, 310905, 1:20, RRID: AB_314840), anti-CD38-PE (Biolegend, 356603, 1:20, AB_2561899), anti-CD40-PE (Biolegend, 334307, 1:20, RRID: AB_1186060), anti-SIGLEC1-APC (Biolegend, 346007, 1:20, RRID: AB_11150773), anti-CD274-PE (Biolegend, 329705, 1:20, RRID: AB_940366), anti-NRP1-PE (Biolegend, 354503, 1:20, RRID: AB_11219200), and anti-SLAMF7 (Santa Cruz, sc-53577, 1:50, RRID: AB_1121905). For the sample incubated with unconjugated anti-SLAMF7, secondary staining employed either anti-mouse-AF647 (**Figure 5C**, Invitrogen, A21236, 1:1,000, RRID: AB_141725) or anti-mouse-AF488 (**Figure S7B**, CST, 4408S, 1:1,000, RRID: AB_10694704) for 1 h at 4°C. All samples were fixed with 4% paraformaldehyde (Biolegend) for 10 min at room temperature, before being analyzed on a Becton-Dickinson FACSCalibur, Becton Dickinson LSR Fortessa or Becton Dickinson Accuri C6. Data was analyzed using FlowJo vX software.

### Immunoblotting

#### Preparation of Cell Lysates for Immunoblot

For whole cell protein analysis, cells were lysed in RIPA buffer (Cell Signaling Technology) containing cOmplete Mini Protease Inhibitor Cocktail (Roche) for 15–30 min at 4°C prior to clarifying by centrifugation at 14,000g for 10 min.

For preparation of samples enriched in PM proteins, cells were stimulated overnight with IFN, then biotinylated and lysed as described for proteomics. PM proteins were enriched by incubation with streptavidin beads for 2 h, followed by washes with lysis buffer and 0.5% SDS. The streptavidin beads were resuspended in SDS page buffer, boiled at 95°C for 10 min and the supernatant collected prior to immunoblot analysis.

#### Immunoblot

Protein concentration was measured using a BCA assay (Pierce) according to the manufacturer’s instructions. Samples were denatured and reduced with 6× protein loading dye (375 mM Tris pH 6.8, 12% SDS, 30% glycerol, 0.6 M DTT, 0.06% bromophenol blue) for 5 min at 95°C. 50 µg of protein was separated by SDS polyacrylamide gel electrophoresis (PAGE) using Mini-PROTEAN TGX precast gels (Bio-Rad, 456-1085), then transferred to polyvinylidene difluoride (PVDF) membranes (0.45 µm pore) using the Bio-Rad wet (**Figures 5E, H** and **Figure S5**) or semi-dry (**Figure 5G**) transfer system. The membrane was blocked with 5% milk in TBST before probing overnight at 4˚C with anti-GAPDH (R&D Systems, MAB5718, 1:10,000, RRID: AB_10892505) and anti-ECE1 (Abcam, ab71829, 1:1,000, RRID: AB_2277809) or anti-phospho-STAT1 (Cell Signaling Technology, 9167S, RRID: AB_561284). Secondary antibodies used were IRDye 680RD goat anti-mouse (LI-COR, 925-68070, 1:10,000, RRID: AB_2651128) and IRDye 800CW goat anti-rabbit (LI-COR, 925-32211, 1:10,000, RRID: AB_2651127). Fluorescent signals were detected using a LI-COR Odyssey CLx, and images were processed using Image Studio Lite version 5.2 (LI-COR).

Revert 700 Total Protein Stain kit (LI-COR) was used to quantify the total abundance of proteins in each of the plasma membrane enriched samples in order to normalize ECE1 signal between unstimulated and IFNα2a-stimulated conditions (**Figure 5H**), according to the manufacturer’s instructions.

### RT-qPCR

Total RNA was extracted using an RNeasy Mini Kit (Qiagen), followed by removal of contaminating DNA using the TURBO DNA-free reagents (Invitrogen) and cDNA synthesis using GoScript Reverse Transcriptase kit (Promega), following the manufacturer’s instructions. RT-qPCR was performed using the TaqMan gene expression master mix (Applied Biosystems) with Taqman probes (ThermoFisher) for ECE1 (Hs01043735_m1), TMEM123 (Hs00920881_m1), and GAPDH (Hs02786624_g1). Analysis was performed on the 7500 Fast & 7500 real-time PCR systems (Applied Biosystems). The PCR program consisted of activation at 95°C for 2 min, followed by 40 cycles of denaturation at 95°C for 5 s and annealing/extension at 60°C for 30 s.

## Results

### Validation of the Workflow in THP-1 Cells

To establish a robust protocol for cellular stimulation with IFNα2a followed by PM protein isolation, we first examined the cultured monocytic cell line THP-1. Cells were cultured overnight in the presence or absence of 1,000 IU/ml IFNα2a then subjected to PM profiling (27, 28). Of 570 proteins with a gene ontology (GO) annotation of “plasma membrane”, “cell surface”, “extracellular” or “short GO” (29), 31 were upregulated >1.5 fold (**Figure S1**, **Table S1**). These included multiple class I major histocompatibility complex (MHC) molecules, the IFN stimulated HIV restriction factor tetherin (Bone Marrow Stromal Cell Antigen 2, BST2), and the receptor tyrosine kinase AXL, all of which are known positive controls (48, 49). A smaller subset of 13 proteins were downregulated >1.5 fold, including the Interferon alpha/beta receptor 1 (IFNAR1) component of the IFN receptor, as has also been previously reported (50).

### Quantitative Comparison of the Cell Surface Proteome of Primary CD14+ Monocytes and CD4+ T Cells

**Figure 1:**
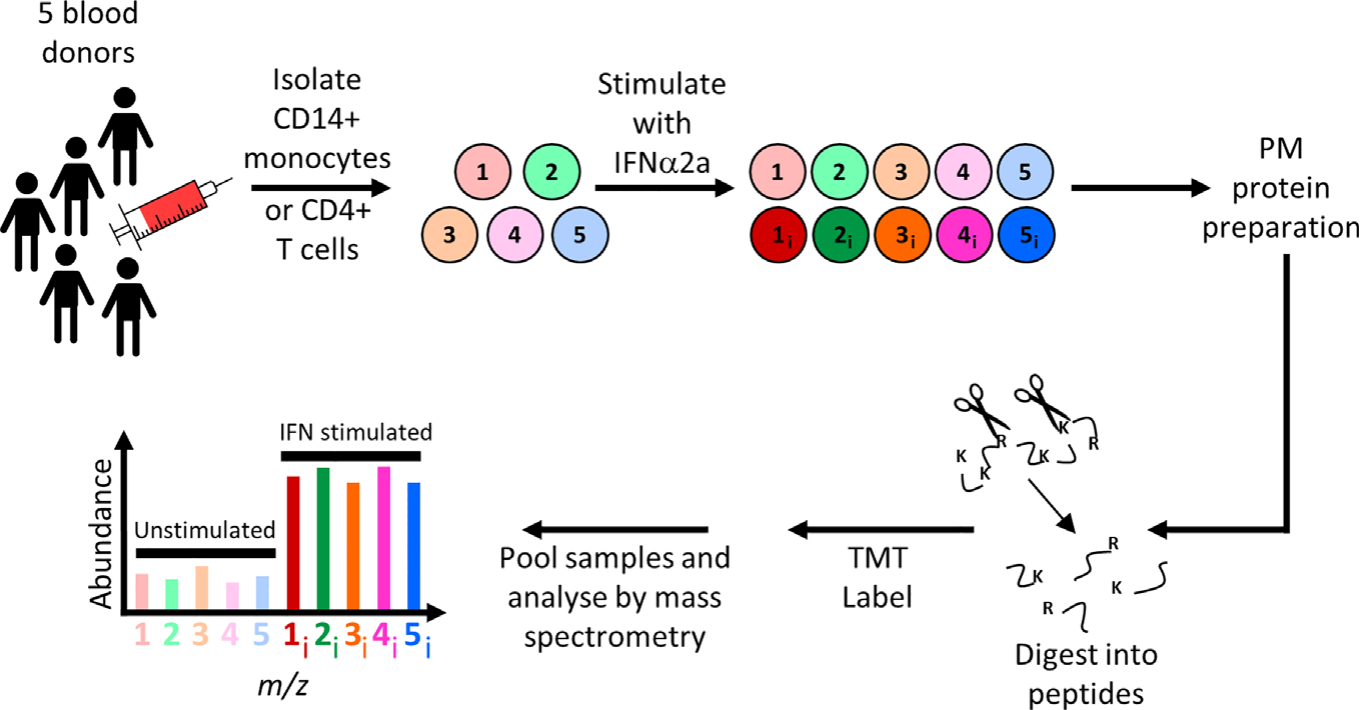
Schematic of experimental workflow for plasma membrane proteomic analysis of IFNα2a-stimulated and unstimulated primary CD14+ monocytes or CD4+ T cells. For each cell type, five independent donations were cultured overnight in the presence or absence of IFNα2a, before selective oxidation and aminooxy-biotinylation of cell surface glycoproteins. Proteins were enriched on streptavidin beads, digested with trypsin, and peptides from each of the 10 samples labeled with tandem mass tag (TMT) reagents then subjected to MS3 mass spectrometry.

To quantify IFNα-stimulated changes in primary leukocytes, peripheral blood mononuclear cells (PBMCs) were isolated from ten donors. Negative selection was used to enrich CD14+ monocytes from five donations, and CD4+ T cells from another five donations. Flow cytometry confirmed that the purity of each population was ≥90% (**Table S2A**). Aliquots of cells from each donation were then either stimulated overnight with IFNα2a or left unstimulated, followed by isolation and quantitation of PM proteins (**Figure 1**). Overall 606 annotated PM proteins were quantified in CD14+ monocytes and 482 in CD4+ T cells (**Table S2B**). All data are shown in **Table S1**, in which the interactive “Plotter” worksheet displays results for each protein of interest.

**Figure 2:**
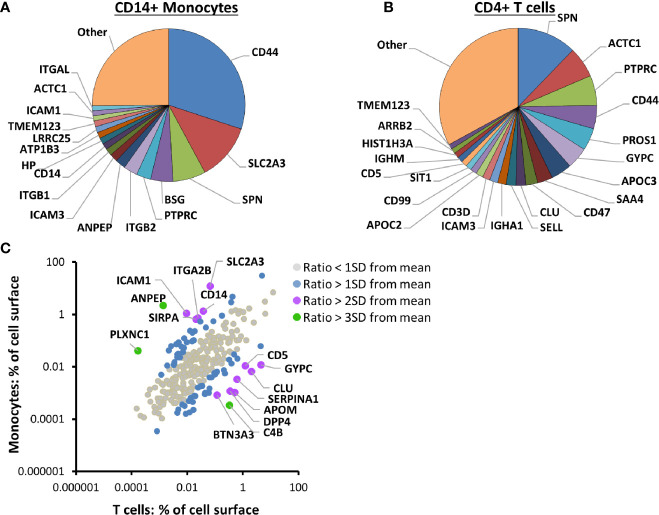
Proteomic cell surface map of primary CD14+ monocytes and CD4+ T cells. **(A)** Pie chart of the relative contribution of individual proteins to the plasma membrane (PM) proteome of unstimulated primary CD14+ monocytes. iBAQ abundance values for each protein were estimated from the sum of the maximum precursor intensity for all contributing peptides divided by the theoretical number of tryptic peptides between 7–30 amino acids in length. The value was scaled according to the signal detected in the five unstimulated samples compared to the total signal in all ten IFN-stimulated and unstimulated samples, to give an estimate of the protein expression at the surface of unstimulated cells. Classical class I and II MHC molecules were excluded from all iBAQ analyses to eliminate bias introduced by differentially expressed alleles between different donors. Proteins contributing less than one percent are grouped into ‘other’. The complete dataset is given in **Tables S1** and **S3A**. **(B)** Pie chart of the relative contribution of individual proteins to the PM proteome of unstimulated primary CD4+ T cells, calculated and displayed as described in **(A)**. The complete dataset is given in **Tables S1** and **S3B**. **(C)** Comparison of the contribution of 280 proteins to the PM proteome of unstimulated primary CD14+ monocytes and CD4+ T cells. Classical class I and II MHC molecules were excluded as described in **(A)**. Color coding illustrates the number of standard deviations (SD) from the mean for each protein’s log_2_ ratio (% PM proteome (CD14+ monocytes)/% PM proteome (CD4+ T cells)). The complete dataset is given in **Tables S1** and **S3C**.

In addition to enabling quantitation of IFNα-stimulated changes in PM proteins, this data facilitated comparison of the cell surface proteome between the different cell types, using a method based on intensity based absolute quantification (iBAQ (25)). Remarkably, 17 proteins contributed more than 1% each to the cell surface proteome of unstimulated CD14+ monocytes, with the summed contribution from all 17 totalling ~75% (**Figure 2A**, **Table S3A**). In fact, the five most abundant proteins composed ~57% of the surface proteome: CD44, solute carrier family 2 facilitated glucose transporter member 3 (SLC2A3), leukosialin (SPN), basigin (BSG) and Protein Tyrosine Phosphatase Receptor Type C (PTPRC). Similarly, in CD4+ T cells, 22 proteins contributed more than 1% of the cell surface proteome, with the summed contribution from all 22 totalling ~67% (**Figure 2B**, **Table S3B**).

The abundance of 280 PM proteins quantified in both CD4+ T cells and CD14+ monocytes generally correlated well, with CD44, SPN (CD43) and PTPRC (CD45) among the five most abundant proteins in both cell types (**Figure 2C**, **Table S3C**). All three molecules have roles in cell adhesion and/or cellular activation (51–54). Expression of other proteins was more cell-type specific. For example, the myeloid-lineage membrane receptor signal regulatory protein alpha (SIRPA) was ~32-fold more abundant on CD14+ monocytes than CD4+ T cells, and the scavenger receptor cysteine-rich superfamily member CD5 was >100 fold more abundant on CD4+ T cells than CD14+ monocytes. SLC2A3 (also known as glucose transporter GLUT-3), was previously found to be 8.4 times more abundant in monocytes compared to lymphocytes, while GLUT1 (SLC2A1) was more abundant in lymphocytes (55), in keeping with the data presented here (**Table S3C**).

The use of multiplexed proteomics also enabled the assessment of donor-to-donor variation in protein expression in unstimulated cells. Both CD14+ monocytes and CD4+ T cells revealed remarkably invariant cell surface proteomes, with a strong positive correlation observed for all pairwise comparisons (**Figure S2A**, **Tables S3A, B**).

### IFNα2a-Stimulated Cell Surface Changes in CD14+ Monocytes

**Figure 3:**
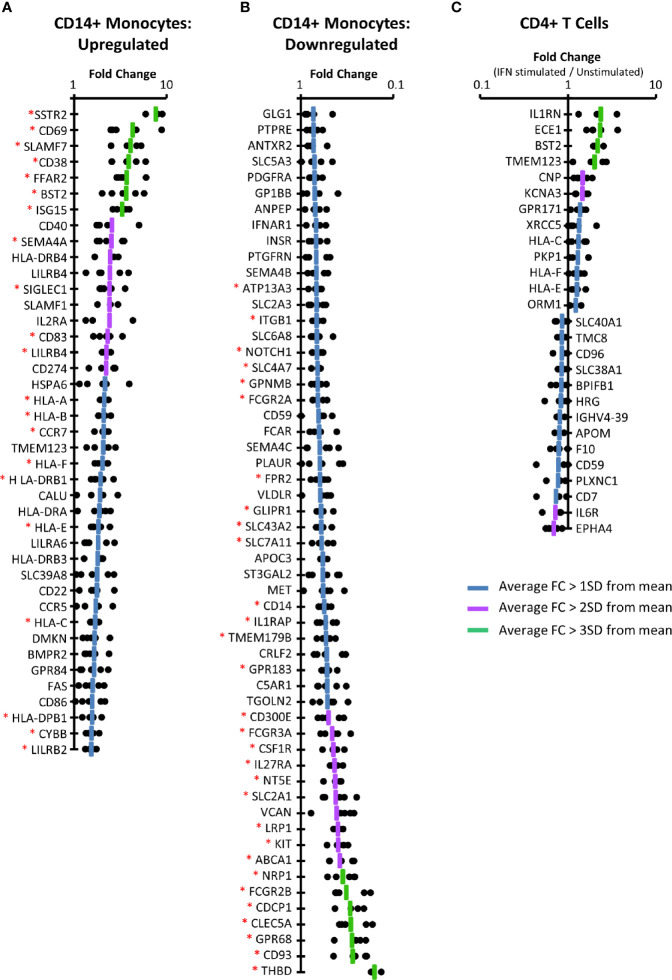
IFNα2a-induced changes at the PM of CD14+ monocytes and CD4+ T cells. **(A)** Proteins consistently upregulated by IFNα2a in primary CD14+ monocytes (FC > 1SD from the mean, with FC > 1 in all donors). Dots display the fold change for each donor, and the line represents average FC. Data for all proteins upregulated >1 SD above the mean, prior to filtering for consistent upregulation is shown in **Figure S3**. A Benjamini-Hochberg-corrected paired, two-tailed t-test was used to estimate the p-value that each protein exhibited significant change in expression upon IFNα stimulation (*p<0.05). The complete dataset is given in **Tables S1** and **S4B**. **(B)** Proteins consistently downregulated by IFNα2a stimulation in primary monocytes, determined as in **(A)** (FC > 1SD from the mean, and FC<1 in all donors). The complete dataset is given in **Tables S1** and **S4C**. **(C)** Proteins consistently up- or downregulated in primary CD4+ T cells, as described in **(A, B)**. The complete dataset is given in **Tables S1** and **S4F, G**.

In primary CD14+ monocytes, 57 proteins were upregulated by >1 SD above the mean protein fold change (FC), of which 41/57 (72%) were consistently upregulated (FC>1) for all donors in which they were quantified (**Figure 3A**, **Figure S3** and **Tables S4A, B** **).** For 21/41 (51%) of these proteins the difference between protein expression in stimulated and unstimulated samples was significant at p < 0.05 (paired two-tailed Student’s t-test, corrected for multiple hypothesis testing using the method of Benjamini-Hochberg (44)). These included several factors well known to exhibit IFNα2a stimulation such as BST2, multiple HLA molecules, and the ubiquitin-like protein ISG15 (56). Extracellular proteins bound to the plasma membrane such as ISG15 were routinely quantified in our plasma membrane preparations. Using the same criteria, 55 proteins were consistently downregulated in CD14+ monocytes, of which 30 exhibited significance with p<0.05 (**Figure 3B** and **Tables S4A, C**).

To validate these findings in independently derived samples, whole monocyte cell populations were enriched from two further donors using a ‘pan-monocyte’ Dynabead enrichment kit. 81% of proteins consistently upregulated by IFNα in CD14+ monocytes were also consistently upregulated in the whole monocyte populations by the same criteria, providing confidence in our data (**Figure S4A**). Many of the proteins most substantially downregulated by IFNα2a in the CD14+ monocytes were similarly downregulated in the whole monocyte population (**Figure S4A**, **Table S4D**).

Finally, comparison of the CD14+ monocyte data to the THP-1 sample identified 357 PM proteins commonly quantified. Twenty-eight proteins were upregulated and 40 downregulated in primary CD14+ monocytes (according to criteria detailed above), whereas 36 were upregulated and 18 downregulated in THP-1s. Of these proteins, 16 were commonly upregulated and six commonly downregulated in both cell types (**Figure S4B**, **Table S4E**). Characterizing the similarities and differences between these cell types is important for evaluating the potential of THP-1s as a model for primary cells.

### IFNα2a-Stimulated Cell Surface Changes in CD4+ T Cells

Applying the same filtering criteria used for analysis of monocyte populations, only 13 proteins were consistently up-regulated and 14 proteins consistently down-regulated in primary CD4+ T cells (**Figure 3C** and **Tables S4A, F, G**). None of these changes were statistically significant, despite consistent regulation among all five donors. This may reflect heterogeneity in populations of CD4+ T cells from different donors, or over-stringency of the multiple testing correction in the context of proteomic data (57). Nevertheless, identification of positive controls again provided confidence in the data, including consistent IFNα2a-stimulated upregulation of multiple HLA molecules and BST2. The IL-1 receptor antagonist (IL1RN) is known to be regulated by IFNα with roles in modulating the inflammatory response (58, 59), and C-type natriuretic peptide (CNP) is a known ISG involved in inhibiting HIV particle assembly (60). Additionally, the IL-6 receptor (IL6R) was downregulated by IFNα, as has been previously been reported (61).

### Comparison of IFNα2a-Stimulated Proteins in Monocytes and T Cells

**Figure 4:**
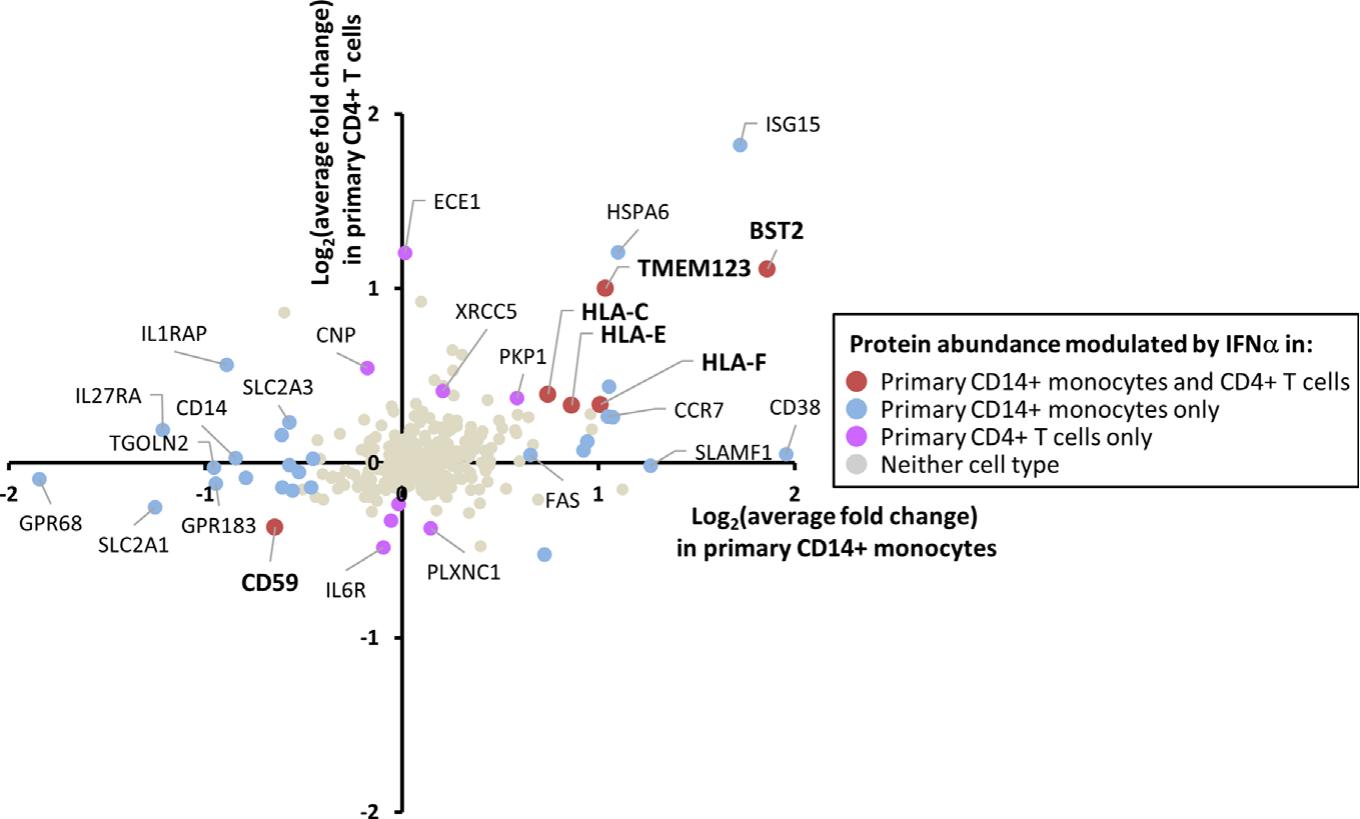
Comparison of the effects of IFNα2a on the plasma membrane (PM) proteome of primary CD14+ monocytes and CD4+ T cells. Comparison of data for 284 proteins quantified in both CD14+ monocytes and CD4+ T cells. These 284 proteins include 280 proteins described in **Figure 2C** and additionally classical class I and class II HLA molecules. Proteins are defined as modulated by IFNα2a according to the previously defined criteria (FC > 1SD from the mean, and FC > 1 in all donors for upregulation, FC < 1 in all donors for downregulation). Full data is shown in **Tables S1** and **S4H**. Certain proteins including ISG15 did not meet cut-offs for upregulation by IFNα2a in CD4+ T cells due to inconsistent upregulation across donors (**Table S1**).

Expression of the IFNAR1 chain of the IFN receptor was detected in monocytes by proteomics (**Table S1**), and expression of IFNAR2 was confirmed in both primary monocytes and T cells by cell surface flow cytometry (**Figure S5A**). Phosphorylation of STAT1 upon IFN stimulation was confirmed in both cell types by immunoblot (**Figure S5B**). However, IFN responses are known to be cell type specific (6, 7). Of the 284 proteins quantified in both CD14+ monocytes and CD4+ T cells, five were IFNα2a-stimulated in both cell types (**Figure 4**). These included the known HIV restriction factor BST2, HLA-C, E and F, and Transmembrane Protein 123 (TMEM123). TMEM123 was also stimulated by IFNα2a in samples from both pan-monocyte donors, and in THP-1 cells (**Figure S4**). In contrast, four proteins were only stimulated by IFNα2a in CD4+ T cells including Endothelin Converting Enzyme-1 (ECE1). 11 proteins were only IFNα2a-stimulated in CD14+ monocytes including ADP-Ribosyl Cyclase 1 (CD38) (**Figure 4**, **Table S4H**). These findings were consistent with the greater breadth of protein changes in CD14+ monocytes compared to CD4+ T cells (**Figure 3**). In keeping with these observations, a previous transcriptomic study demonstrated that in the context of TNFα pre-treatment, subsequent IFNβ treatment stimulated many more transcripts in monocytes than in T cells (667 monocyte-specific transcripts compared to 21 T cell-specific transcripts) (62). Additionally, combined data from multiple transcriptomic studies of primary and cultured cells stimulated with type I IFNs demonstrated 567 ISGs changing in abundance upon IFN stimulation for monocytes compared to 124 for T cells (7).

### Donor-to-Donor Variation in IFNα2a–Stimulated Changes in the PM Proteome

In contrast to a relative invariance in the cell surface proteome between different donors (**Figure S2A**), there was a greater degree of donor-to-donor variation in IFNα2a-induced changes in the PM proteome, particularly for CD4+ T cells (**Figure S2B**). While just 13 proteins met criteria for consistent upregulation in T cells, 112 proteins were upregulated more than 1.5 fold in at least one donor. Importantly, this variation did not result from a systematically greater IFNα2a effect in some donors than other, as the pattern of induced FCs appeared random (**Figure S6**).

### Validation of IFNα2a-Stimulated Changes

**Figure 5:**
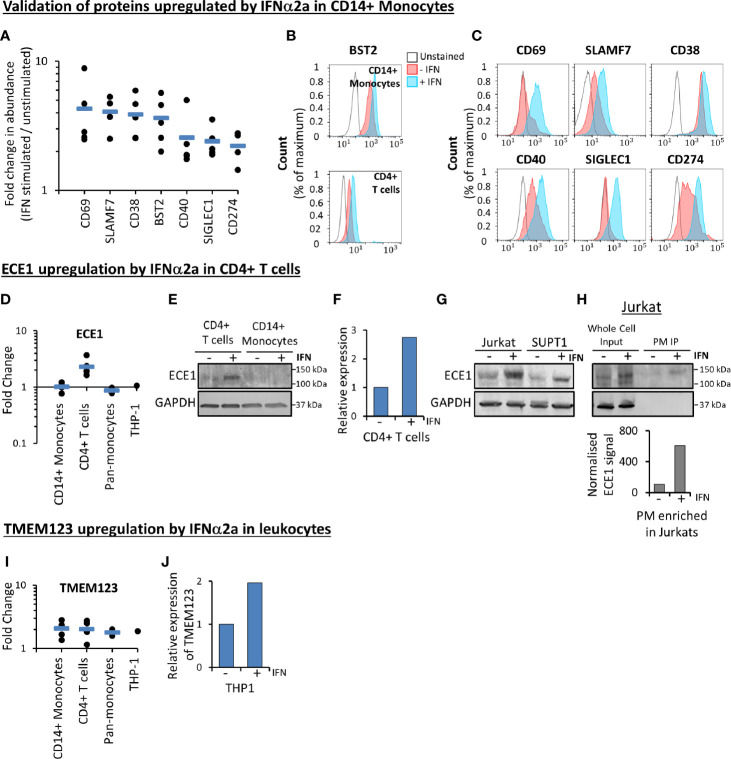
Validation of proteomic data. **(A)** Proteomic quantitation of a subset of proteins stimulated by IFNα2a in primary CD14+ monocytes. Each point represents a single donor. Data is a subset of that shown in **Figure 3A**, and **Table S1**. **(B)** Flow cytometry-based validation of IFNα2a-stimulated change in BST2 expression at the surface of primary CD14+ monocytes (top) and CD4+ T cells (bottom). Similar data from an additional donor is shown in **Figure S7A**. **(C)** Validation by flow cytometry of a selection of proteins stimulated by IFNα2a in primary CD14+ monocytes. Early activation antigen CD69, CD38, CD40, sialoadhesin SIGLEC1 and programmed cell death 1 ligand 1 (CD274) were quantified in CD14+ monocytes from one donor, while SLAMF7 was quantified in cells donated by a second individual. Red (unstimulated) and blue (stimulated with IFNα2a) coloring as in **Figure 5B**. For SLAMF7 staining, the gray line represents a control sample stained only with secondary antibody (anti-mouse-AF647). For all other samples, the gray line represents unstained samples as directly conjugated antibodies were employed. Similar data from two additional donors is shown in **Figures S7B** **, (C). (D)** Proteomic quantitation of cell surface ECE1 in different cell types. Full data is given in **Table S1**. **(E)** ECE1 protein is upregulated in the whole cell protein lysate from IFNα2a-stimulated primary CD4+ T cells. Data is from a single donor. **(F)** qPCR confirming IFNα2a-stimulated upregulation of ECE1 in primary CD4+ T cells, at the level of mRNA (Data is from a single donor, with three technical replicates). **(G)** Immunoblot confirming IFNα2a-stimulated upregulation of ECE1 in whole cell protein lysates from two cultured T cell lines Jurkats and SUPT1s (n=1). **(H)** Immunoblot after immunoprecipitation (IP) of biotinylated cell surface proteins confirming IFNα2a-stimulated upregulation of ECE1 at the cell surface of Jurkat T cells. ‘Whole cell input’ represents protein lysates prior to PM enrichment, and ‘PM IP’ represents PM-enriched samples. For the quantitation shown in the bar chart below the immunoblot, signals from the ECE1 bands following immunoprecipitation were normalized according to the relative amounts of PM protein loaded on both lanes of the gel. This was measured using a total protein stain and quantified using Image Studio Lite (n=1). **(I)** Proteomic quantitation of cell surface TMEM123, demonstrating upregulation by IFNα2a in all cell types. Full data is given in **Table S1**. **(J)** TMEM123 upregulation by IFNα2a was validated in THP-1s by qPCR (three technical replicates).

A subset of proteins modulated by IFNα2a in primary CD14+ monocytes were selected for validation in three donors using flow cytometry (**Figures 5A–C** and **Figure S7**). ECE1 was the most substantially IFNα2a-stimulated cell surface protein in all five donors in primary CD4+ T cells (**Figure 3C**). This change was validated at the level of whole cell proteins and transcript (**Figures 5D–G**). Furthermore, stimulation of ECE1 at the cell surface of Jurkat T cells was demonstrated by immunoprecipitation of biotinylated cell surface proteins and immunoblot (**Figure 5H**). Apart from BST2 and HLA molecules, TMEM123 was the only protein upregulated in both monocytes and T cells (**Figure 4**). Stimulation of TMEM123 transcript in THP-1 cells by IFNα2a was validated (**Figures 5I, J**). However, despite testing four commercially available antibodies for TMEM123 by immunoblot and flow cytometry in cells depleted of TMEM123 by RNAi or overexpressing TMEM123, we were unable to identify any reagent that exhibited specific binding to TMEM123 protein meaning that validation at the protein level was not possible.

## Discussion

This study represents the first systematic analysis of the effects of type I IFN at the cell surface, and the most comprehensive analysis of the surface proteome of primary monocytes and CD4+ T cells to date. 606 and 482 annotated cell surface proteins were quantified in primary CD14+ monocytes and CD4+ T cells respectively. For comparison, only 229 proteins were previously identified at the surface of CD4+ T cells by a combination of proteomics and flow cytometry, and 274 proteins from CD4+CD25- cells by proteomics (63, 64). Furthermore, the use of TMT technology enabled multiplexing of samples in order to investigate samples from five donors in parallel, allowing direct investigation of donor-to-donor variation in protein abundance, identification of consistent IFNα2a-stimulated effects and quantitation of cellular responses to IFNα2a.

Whereas just 13 proteins were consistently upregulated by IFNα2a at the PM of CD4+ T cells, 41 were upregulated in CD14+ monocytes. Some of these proteins are maturation markers of monocyte-derived DCs, including CD83 (65), CD38 (66), the co-stimulatory molecule CD86, C-C Chemokine Receptor Type 7 (CCR7) and HLA-DR (67). Mature DCs are routinely generated *in vitro* by treatment of monocytes with GM-CSF, IL-4 and TNFα. In a previous comparative microarray analysis, GM-CSF/IL-4/TNFα derived DCs expressed higher levels of transcripts involved in phagocytosis and adhesion, whereas DCs generated by stimulation with GM-CSF and IFNα expressed greater levels of transcripts associated with migration (68). CD86, HLA-DR, and CCR7 transcripts were upregulated in GM-CSF/IFNα-generated DCs, consistent with our observations. Similarly, a limited flow cytometry-based study of IFNα stimulation identified upregulation of CD86, CD83 and CCR7 in monocyte and whole PBMC populations (69). Our study thus suggests that stimulation with IFNα2a alone may be sufficient to induce a degree of monocyte differentiation, and that cellular differentiation may explain the broader cell surface regulation of a subset of the proteins modulated by IFNα2a.

A particularly interesting use of our data may be the identification of novel cell surface antiviral restriction factors, on the basis of both stimulation by IFN and targeting for degradation by a viral factor (5). For example, BST2 (tetherin) was upregulated by IFNα2a on CD14+ monocytes and CD4+ T cells. BST2 has been extensively characterized for its ability to inhibit replication of HIV in addition to flaviviruses, herpesviruses, rhabdoviruses, paramyxoviruses and arenaviruses, in part by impeding viral exit from the cell, and by stimulating signaling that leads to NF-kB activation (48, 70–72). BST2 is antagonized by the HIV protein Vpu, the Kaposi’s sarcoma herpesvirus (KSHV) K5 protein and Ebola virus glycoprotein (48, 73, 74). In CD4+ T cells, excluding MHC molecules, only 10 proteins were stimulated by IFNα2a at the cell surface. In addition to BST2, CNP also has antiviral activity (60), suggesting that the other eight IFNα2a-stimulated CD4+ T cell proteins might be particularly enriched in novel cell surface antiviral factors. ECE1 was one of the most highly upregulated proteins quantified in all five CD4+ T cell donations, and was not upregulated in monocytes. We previously determined that ECE1 is downregulated by the human cytomegalovirus US2 protein (28, 75, 76) and is modestly downregulated at the plasma membrane during infection with HIV (77). Further investigation will be required to determine whether ECE1 has the ability to restrict viruses. ECE1 is a zinc metalloendopeptidase, with the primary function of cleavage of endothelin to its mature vasoactive form. It is also able to cleave other peptides (78), and localizes to endosomes in addition to the plasma membrane, where it has roles in receptor recycling and re-sensitisation for substance P and TLR9 (79–81). Potential mechanisms of viral restriction by this molecule might thus include cleavage of viral proteins or host proteins necessary for infection, or alternatively roles in stimulating host immunity.

Other than HLAs and BST2, TMEM123 was the only protein consistently upregulated in both primary monocytes and CD4+ T cells by IFNα2a. This might provide a novel pan-leukocyte marker for IFNα2a stimulation in addition to another candidate antiviral factor. TMEM123 has so far been poorly characterized; also known as Porimin, it was originally identified due to the ability of anti-porimin antibody to stimulate oncotic cell death in Jurkat T cells (82, 83). Generation of new resources to further validate this finding at the protein level will be a necessary first step in future investigations.

TMEM123, BST2, and HLA molecules were some of the most consistently IFN-stimulated proteins, both between cell types and also between donors. While cell surface proteomes were remarkably invariant between different donors, much greater variability was observed between donor leukocytes in responses to IFNα2a stimulation, particularly in CD4+ T cells. Possible explanations for this phenomenon include (a) heterogeneity in the CD4+ T cell populations examined between different donors (84); (b) differential activation of STAT proteins and IFN signaling pathways between donors, or polymorphisms in the promotors or coding regions of IRFs, JAK or STAT proteins (6); or (c) a lack of substantial IFNα2a response in a large proportion of CD4+ T cell proteins meaning that many proteins studied exhibited relatively small changes in expression. Although in this investigation our focus was on identifying protein changes that were most consistent between different donors, it may nevertheless be valuable to investigate proteins that exhibit more variable inter-donor changes. Side effects and efficacy of IFN-based therapies for viral hepatitis and cancers are known to vary between patients, which may partly be explained by differences in cellular responses to treatment (85–87). A larger study would be required to comprehensively characterize the true extent of donor-to-donor variability for such molecules.

An alternative strategy for identifying the most biologically important molecules that are stimulated by IFNα2a could combine our measurements of cell surface protein abundance and IFNα2a-stimulated fold change. For example, intracellular adhesion molecule 1 (ICAM1) did not meet criteria for IFNα2a stimulation in CD14+ monocytes, only exhibiting an average 1.27-fold change in comparison to unstimulated cells. However, as a highly abundant protein at the cell surface, accounting for 1.1% of cell surface molecules in CD14+ monocytes, the change in number of ICAM1 molecules at the cell surface upon IFNα2a stimulation would be considerably greater than other proteins of low abundance but exhibiting higher absolute IFN-simulated fold changes (for example, SIGLEC-1, 2.4-fold change, 0.0007% of CD14+ monocyte cell surface). Such analysis could highlight a different set of IFNα2a-stimulated proteins.

In this study, we have provided a comprehensive map of the cell surface of resting and IFNα2a-stimulated primary leukocytes from multiple donors, complementing and substantially extending previous transcriptomic studies. Understanding how IFNα2a differentially modulates the surface of immune cells will enable a more complete understanding of the IFN response, identifying candidate cellular antiviral factors, and factors which may predict individual responses to IFN-based therapies.

## Data Availability Statement

The mass spectrometry proteomics data have been deposited to the ProteomeXchange Consortium (http://www.proteomexchange.org/) via the PRIDE partner repository with the dataset identifier PXD022834. All materials described in this manuscript, and any further details of protocols employed can be obtained on request from the corresponding author by email to mpw1001@cam.ac.uk.

## Ethics Statement

The studies involving human participants were reviewed and approved by the University of Cambridge Human Biology Research Ethics Committee. The patients/participants provided their written informed consent to participate in this study.

## Author Contributions

LS, MP, LH, and MPW designed the research. LS, MP, LH, JW, and RA performed the experiments. LS, BR, JH, and MPW analyzed the proteomic data. LS and MPW wrote the manuscript. LS, MP, LH, BR, MRW, NM, and MPW edited the manuscript. MPW supervised all research. All authors contributed to the article and approved the submitted version.

## Funding

This work was supported by a Wellcome Trust Senior Clinical Research Fellowship (108070/Z/15/Z) to MPW, Wellcome Trust PhD Studentships to LS (109078/Z/15/Z), MP (203747/Z/16/Z), and LH (220015/Z/19/Z), a Medical Research Council (MRC : UKRI) programme grant (MR/S00081X/1) to MRW, and an MRC Clinician Scientist Fellowship (CSF MR/P008801/1) and NHSBT workpackage (WPA15-02) to NM. This study was additionally supported by the Cambridge Biomedical Research Centre, UK.

## Conflict of Interest

The authors declare that the research was conducted in the absence of any commercial or financial relationships that could be construed as a potential conflict of interest.
