# Supplementary material for: Comparative Cell Surface Proteomic Analysis of the Primary Human T Cell and Monocyte Responses to Type I Interferon
Source: Front Immunol. 2021 Feb 8;12:600056. doi: 10.3389/fimmu.2021.600056 (PMC7897682; doi:10.3389/fimmu.2021.600056)
Supplement: Supplementary file 1 [file DataSheet_1.pdf]

# *Supplementary Material*

## SUPPLEMENTARY FIGURES

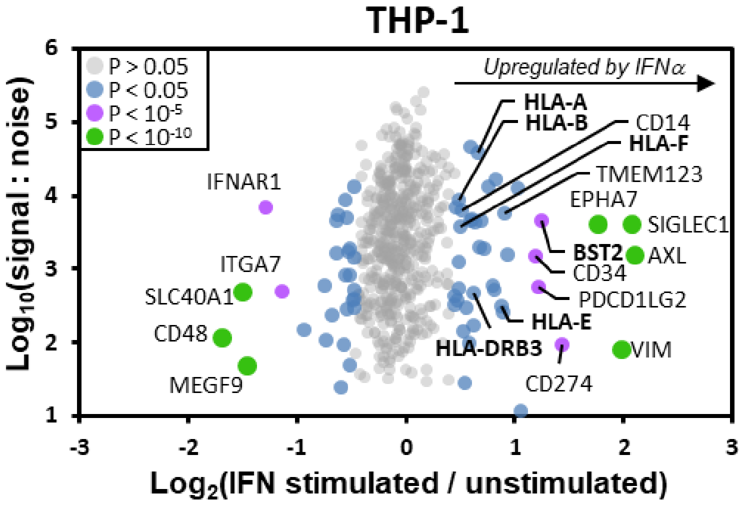


***Supplementary Figure S1. Scatter plot of IFNα2a induced changes in 570 annotated PM proteins quantified in THP-1 cells.*** Benjamini-Hochberg (BH)-corrected significance B was used to estimate p-values (45). The positive controls HLA and BST2 are highlighted in bold.  The complete dataset is given in **Table S1.**

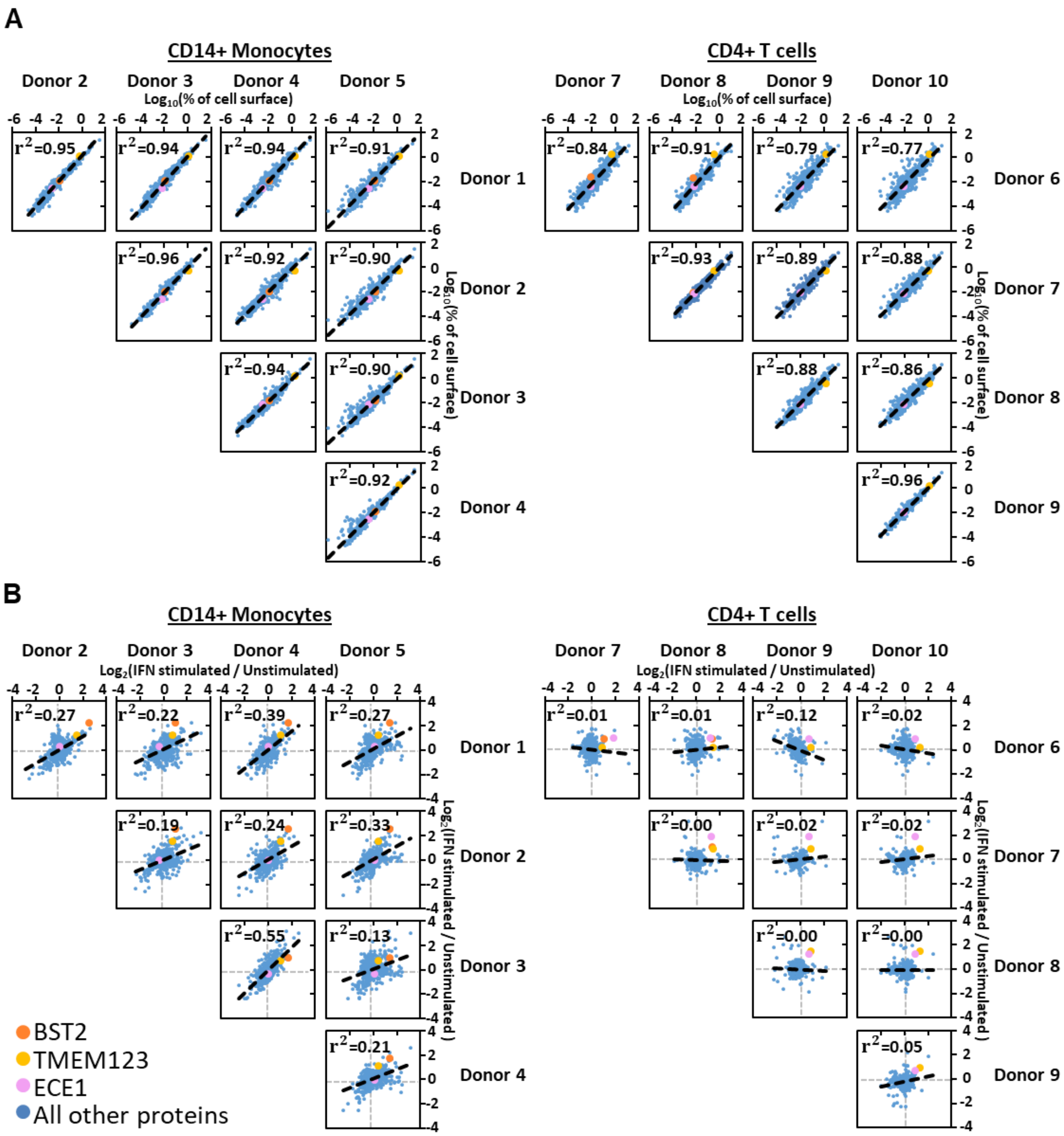


***Supplementary Figure S2. Variation of cell surface proteomes and IFNα2a induced changes between donors***

(A) Comparison of the relative contribution of each protein to unstimulated cell surface proteomes for each of five donors, calculated as described in Figure 2A. Classical class I and II MHC molecules were excluded from this analysis. The complete dataset is given in **Table S1** and **Table S3**.

(B) Comparison of the IFNα2a-stimulated change in expression of each protein between donors. The complete dataset is given in **Table S1**.

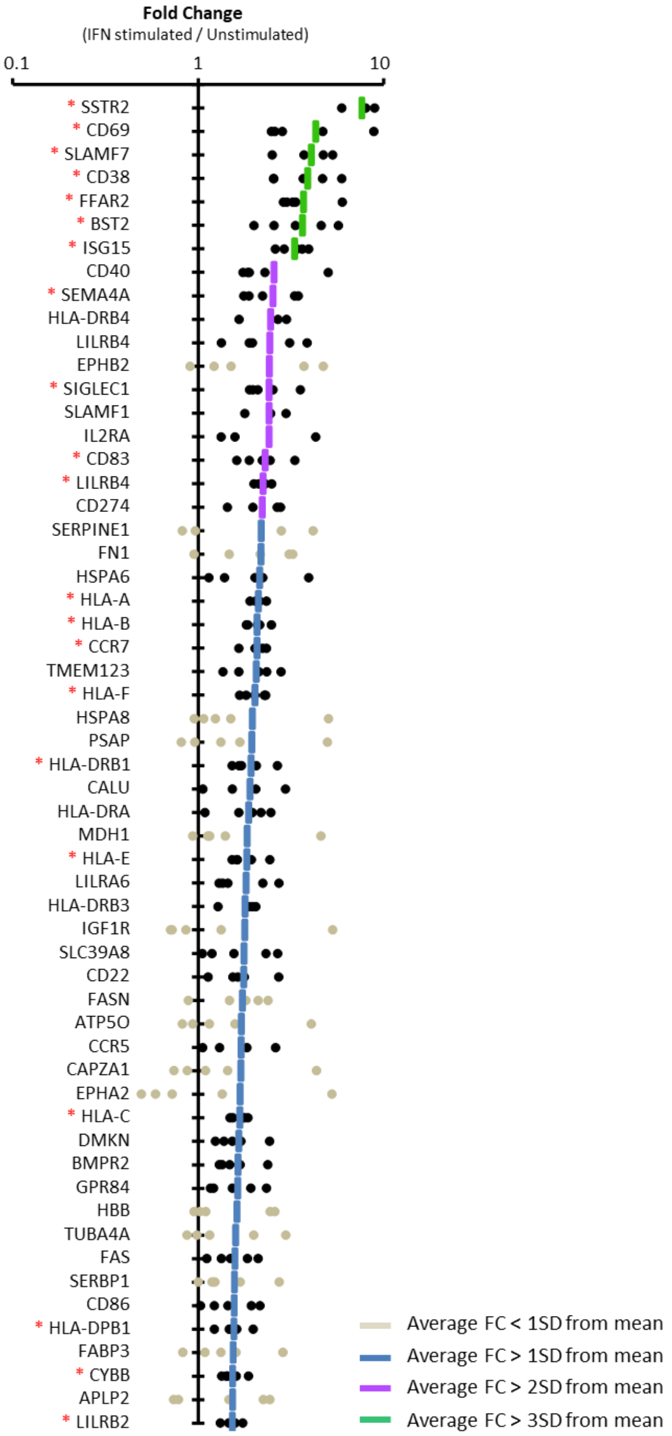


***Supplementary Figure S3. IFNα2a induced changes in CD14+ monocytes.*** Proteins upregulated by >1 SD above the mean protein FC by IFNα2a in primary CD14+ monocytes. To obtain data displayed in Figure 3, proteins that were not consistently upregulated in all donors (grey dots) were excluded. A Benjamini-Hochberg corrected paired, two-tailed t-test was used to estimate the p-value that each protein exhibited significant change in expression upon IFNα stimulation (*p<0.05). The complete dataset is given in **Table S1**.

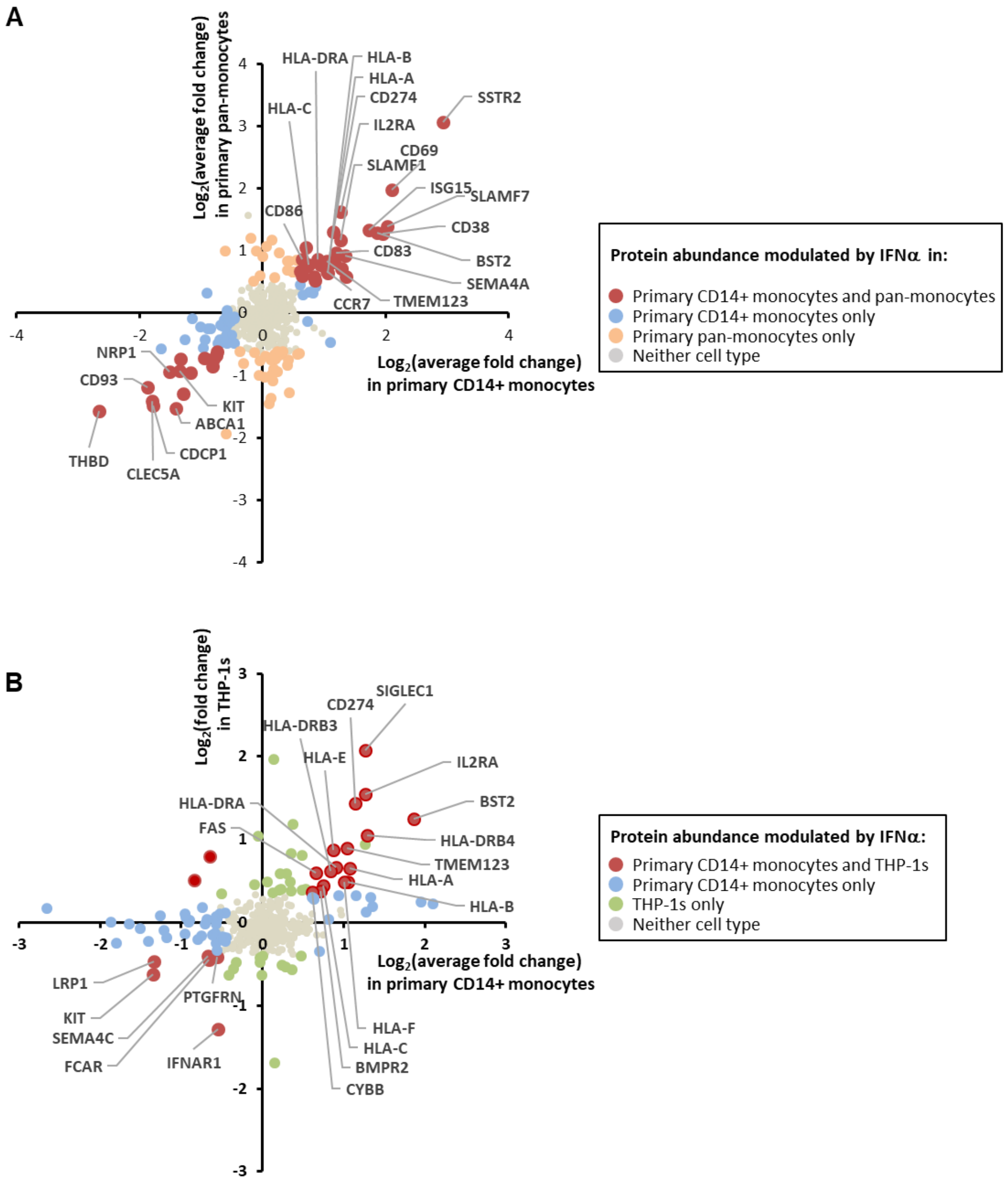


***Supplementary Figure S4. IFNα2a-induced changes in primary monocytes and THP-1 cells.***

(A) IFNα2a induced changes in primary CD14+ monocytes from five donors compared to changes in samples of whole monocyte populations from two additional donors. Proteins were defined as being modulated by IFNα2a if they met the previously described criteria (FC > 1 SD from the mean, and for upregulation FC > 1 in all donors; for downregulation, FC <1 in all donors). The complete dataset is given in **Table S1** and **Table S4D**.

(B) IFNα2a-induced changes in primary CD14+ monocytes compared to those observed in the cultured monocyte cell line THP-1. Criteria for modulation by IFNα2a were as described in (A) for CD14+ monocytes, and >1 SD from average fold change for THP-1 cells to enable comparison using similar criteria. The complete dataset is given in **Table S1** and **Table S4E**.

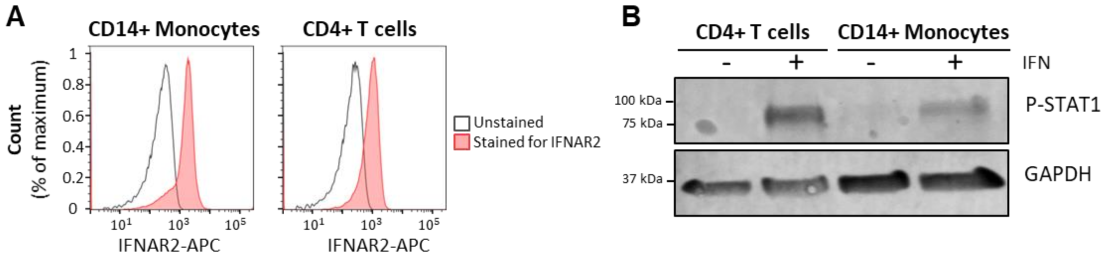


***Supplementary Figure S5. IFNα2a signaling in primary CD14+ monocytes and CD4+ T cells.***

(A) Flow cytometry confirming expression of IFNAR2 at the surface of unstimulated primary CD14+ monocytes (left) and CD4+ T cells (right). For the CD4+ enrichment, CD4 microbeads were used. Therefore the CD3+ population was gated in order to exclude CD4+ CD14+ monocytes that were co-isolated.

(B) Immunoblot confirming phosphorylation of STAT1 in response to IFNα2a stimulation of primary CD14+ monocytes and CD4+ T cells.

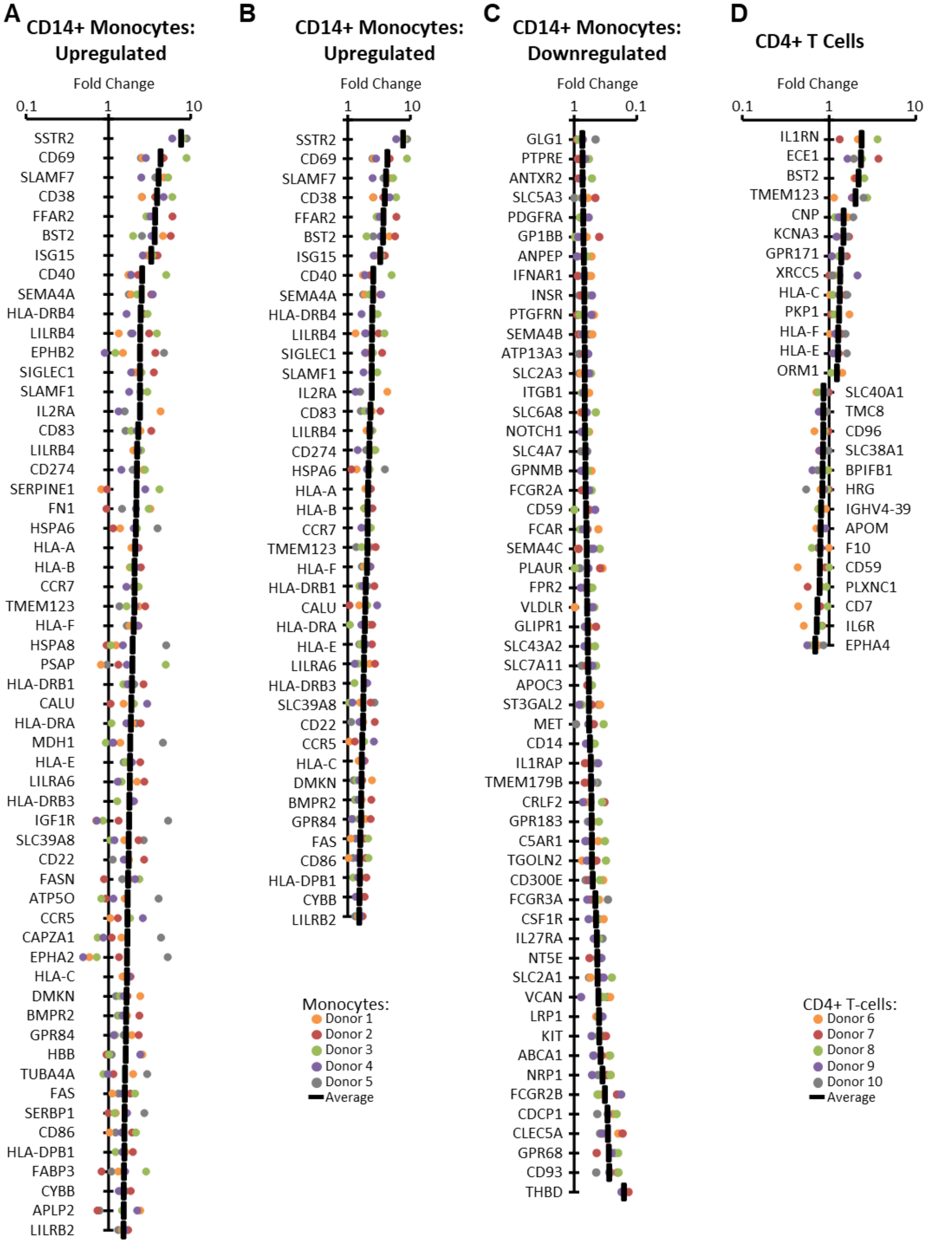


***Supplementary Figure S6. IFNα2a-induced changes identified by individual donors***

(A) Proteins upregulated by IFNα2a in primary CD14+ monocytes, as in Figure S3.

(B) Proteins upregulated by IFNα2a in primary CD14+ monocytes, as in Figure 3A.

(C) Proteins downregulated by IFNα2a in primary CD14+ monocytes, as in Figure 3B.

(D) Proteins up or downregulated by IFNα2a in primary CD4+ T cells, as in Figure 3C.

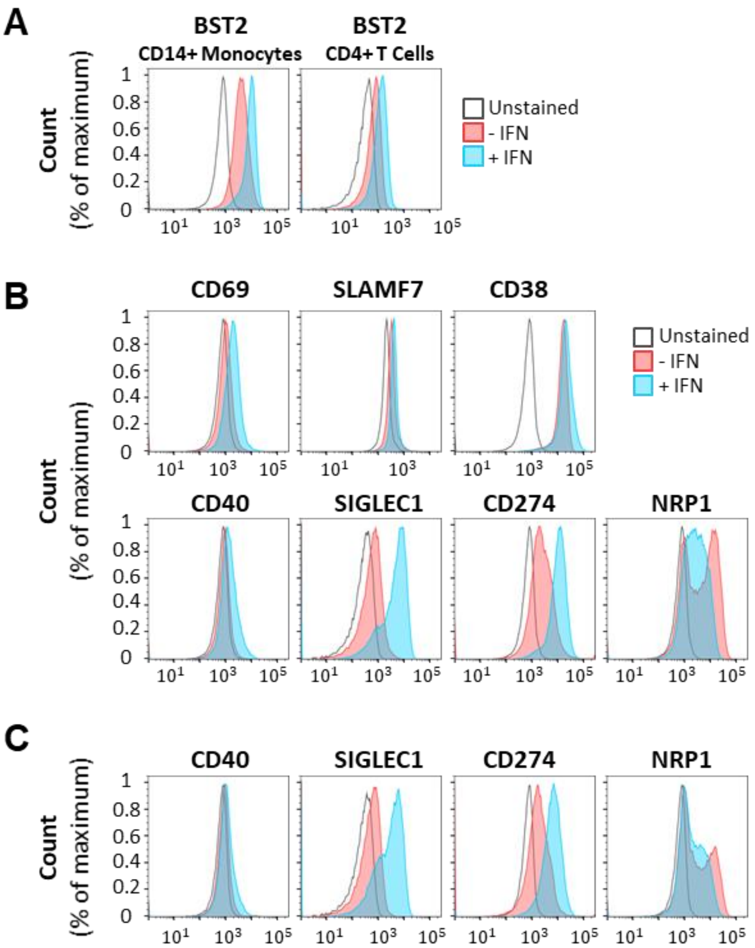


***Supplementary Figure S7. Validation of proteomic data in additional donors***

(A) Flow cytometry-based validation of IFNα2a-stimulated upregulation of BST2 at the surface of primary CD14+ monocytes and CD4+ T cells, from an additional donor (see also Figure 5B).

(B) Validation by flow cytometry of a selection of proteins stimulated by IFNα2a in primary CD14+ monocytes from the same additional donor as (A) (see also Figure 5C). IFNα2a-stimulated downregulation of NRP1 was additionally validated in cells from this donor. The grey line represents unstained samples in all cases.

(C) Validation by flow cytometry of a selection of proteins up-or down-regulated by IFNα2a in primary CD14+ monocytes from a further donor (see also Figure 5C, S7B).

## SUPPLEMENTARY TABLES

**Table S1 (separate .xlsx file).** Interactive spreadsheet of all proteomic data. The ‘plotter’ worksheet enables generation of a graph showing IFN-stimulated protein changes for each cell type. The ‘data’ worksheet shows minimally annotated protein data, for which the only modifications are formatting, deletion of contaminants, normalisation, and summing of the S:N for HLA-A, B, C and DRB1. Additionally, the IFN induced FCs and the cell surface abundance are given in the ‘Data’ worksheet, calculated as described in the methods.

**Table S2 (separate .xlsx file). (A)** Pre- and post- enrichment cell purity for CD14+ monocytes and CD4+ T cells used in proteomic experiments, as assessed by flow cytometry. **(B)** Proteins and peptides quantified by proteomics in each cell type.

**Table S3 (separate .xlsx file).** Abundance of proteins at the unstimulated cell surface of **(A)** primary CD14+ monocytes and **(B)** CD4+ T cells. **(C)** Comparison between the CD14+ monocytes and CD4+ T cells.

**Table S4 (separate .xlsx file).** Proteins meeting criteria for consistent modulation by IFNα in primary cells. **(A)** Number of proteins modulated by IFNα2a in primary CD14+ monocytes and CD4+ T cells. **(B)** Upregulated proteins in primary CD14+ monocytes. **(C)** Downregulated proteins in primary CD14+ monocytes. **(D)** Comparison of proteins modulated by IFN in primary CD14+ and pan monocytes. **(E)** Comparison of proteins modulated by IFN in primary CD14+ monocytes and cultured THP-1s. **(F)** Upregulated proteins in primary CD4+ T cells. **(G)** Downregulated proteins in primary CD4+ T cells. **(H)** Comparison of proteins modulated by IFN in primary CD14+ monocytes and CD4+ T cells.

**Table S5 (separate .xls file).** Details of the proteomic experiments. **(A)** TMT sample labelling. **(B)** Details of mass spectrometry runs for each cell line.
